# Biology, Methodology or Chance? The Degree Distributions of Bipartite Ecological Networks

**DOI:** 10.1371/journal.pone.0017645

**Published:** 2011-03-03

**Authors:** Richard J. Williams

**Affiliations:** Microsoft Research, Cambridge, United Kingdom; German Cancer Research Center, Germany

## Abstract

The distribution of the number of links per species, or degree distribution, is widely used as a summary of the topology of complex networks. Degree distributions have been studied in a range of ecological networks, including both mutualistic bipartite networks of plants and pollinators or seed dispersers and antagonistic bipartite networks of plants and their consumers. The shape of a degree distribution, for example whether it follows an exponential or power-law form, is typically taken to be indicative of the processes structuring the network. The skewed degree distributions of bipartite mutualistic and antagonistic networks are usually assumed to show that ecological or co-evolutionary processes constrain the relative numbers of specialists and generalists in the network. I show that a simple null model based on the principle of maximum entropy cannot be rejected as a model for the degree distributions in most of the 115 bipartite ecological networks tested here. The model requires knowledge of the number of nodes and links in the network, but needs no other ecological information. The model cannot be rejected for 159 (69%) of the 230 degree distributions of the 115 networks tested. It performed equally well on the plant and animal degree distributions, and cannot be rejected for 81 (70%) of the 115 plant distributions and 78 (68%) of the animal distributions. There are consistent differences between the degree distributions of mutualistic and antagonistic networks, suggesting that different processes are constraining these two classes of networks. Fit to the MaxEnt null model is consistently poor among the largest mutualistic networks. Potential ecological and methodological explanations for deviations from the model suggest that spatial and temporal heterogeneity are important drivers of the structure of these large networks.

## Introduction

Describing complex ecosystems as networks of interacting components and explaining the structure of those interaction networks is an essential part of understanding the role of biodiversity in the function and robustness of ecological communities [1], [2]. While food webs, networks of antagonistic consumer-resource interactions, have a long history of study and are the most familiar example of ecological networks [3], [4], [5], significant attention has recently been focused on networks of mutualistic interactions such as plants and their pollinators or plants and seed dispersers [6], [7]. These networks provide a valuable overview of one type of mutualism occurring within a community and several apparently general patterns in the structure of mutualistic networks have been found [8], [9]. Co-evolutionary processes are believed to play a strong role in shaping mutualistic communities [8], [9], though others have questioned whether such processes structure mutualistic networks [10], [11].

The distribution of the number of links per species, or degree distribution, is a widely used summary of the topology of complex networks [12] that has been studied in both food webs and mutualistic networks [9], [13], [14]. Because of its central role in describing network topology, considerable importance has been placed on understanding the processes driving the form of the degree distribution in ecological networks [9], [11], [13], [14]. Interest in the relative abundance of generalists and specialists motivated early studies of networks of mutualistic interactions [15], [16], and such networks were found typically to have strongly skewed degree distributions, with many species with few links and few species with many links [9], [17]. Earlier work found that degree distributions in mutualistic networks are best-fit by a truncated power law [9], but recent work does not support that finding [18]. Similar attempts to fit degree distributions to particular functional forms for food webs have also produced variable results [13], [14], [19]. The obvious difference between the observed skewed distributions and the binomial distributions of random networks [20] has driven the assumption that these skewed distributions are a result of ecological or evolutionary processes shaping species interactions [7], [9].

From early ideas about succession [21], [22] through more recent debates about community assembly [23], [24] to current research into macroecological patterns [25], the debate as to whether perceived patterns in ecosystem properties are the result of chance, biological processes or bias in the data has been an enduring theme in ecological research. The principle of maximum entropy [26] asserts that the least biased probability distribution satisfying a set of constraints is the maximum entropy distribution, and any other distribution would be assuming information not captured by the constraints. It has recently been recognized as a powerful tool in the search for explanations of ecological patterns and has been used to argue that a number of macroecological patterns can be predicted with minimal appeal to specific ecological processes [27], [28]. Recently it was shown that a null model for degree distributions of food webs based on MaxEnt could not be rejected as a null model for 57% of the food web degree distributions studied [29]. This very simple MaxEnt model requires a minimal amount of ecological information: the number of species, the number of species with no prey (basal species) or predators (top species), and the number of links in the network.

Since food webs and mutualistic networks are built primarily from antagonistic and mutualistic interactions respectively, it is interesting to consider whether the different types of interaction causes the structure of these two classes of networks to be significantly different. Mutualistic networks are bipartite networks, with interactions occurring between two groups of species, here plants and animals, but not within the groups. While food webs are not bipartite since they include taxa at many trophic levels and interactions can occur between animals, an obvious subset of a food web, the primary producers and their consumers, form a natural counterpart to the mutualistic plant-animal networks, one in which the interactions are primarily antagonistic. The different types of interaction cause different pressures on organism's behavior, and so it is reasonable to expect networks dominated by antagonistic or mutualistic interactions to have different structure. A study of 14 food webs included as part of a much larger study of mutualistic networks showed that mutualistic and antagonistic networks differed significantly in their nestedness [8]. Given that different ecological processes may shape the networks, it is possible that the degree distributions of these two different types of networks also have different forms.

The goals of this work are three-fold. First, to test whether a MaxEnt model like that used to predict food web degree distributions [29] can predict the degree distributions of mutualistic networks; second, to compare the deviation of mutualistic and antagonistic networks from the MaxEnt model to better understand how the structure of these two classes of networks differs; and third, to explore how specific features of some mutualistic networks might influence their degree distributions and drive them away from the MaxEnt expectation.

## Methods

The degree distributions analyzed are from 68 mutualistic networks compiled for two earlier studies [30], [31] and 47 bipartite networks formed by retaining only the basal taxa (plants and detritus), their consumers and the links between these two groups of taxa from food webs used in an earlier study of food web degree distributions [29]. In these bipartite networks, *S* is the total number of taxa, *S_P_* is the number of plants or basal taxa (some antagonistic networks include detritus as a basal node), *S_A_* is the number of animals or consumers and *L* is the number of connections between these two groups of taxa. The connectivity of a bipartite network *C_B_* = *L*/(*S_A_S_P_*) is the fraction of possible links that occur. Basic properties of these networks are given in table S1. In many food webs, plant nodes are highly aggregated, resulting in a significantly higher fraction of the antagonistic networks have relatively few plant taxa (19 of 47 antagonistic networks have *S_P_*<10; 7 of 51 mutualistic networks have *S_P_*<10, 2-tailed *p* = 0.0032 Fisher's exact test).

None of the bipartite antagonistic networks considered here have more than 134 species. Since network properties are generally dependent on the number of nodes and links in the network [4], [32], similar size mutualistic and antagonistic networks are compared. To avoid comparing very different-sized networks, the mutualistic networks are split into two groups, the 51 networks with less than 135 species which are compared with the similarly sized bipartite antagonistic networks, and a group of 17 large mutualistic networks with *S*>140 that have no counterpart antagonistic networks of a similar scale.

A network's degree distribution is the distribution of the number of links attached to each node in the network. The networks considered here are directed, in that the interactions are asymmetric. In the food webs, one species is a consumer and the other a resource while in the mutualistic network the plant gives up food and receives a reproductive benefit while the animal receives food and transports reproductive material. It is therefore useful to consider the degree distribution of each group of nodes in the bipartite network separately. The distribution of the number of links connected to the plant or resource species is called the plant distribution while the distribution of the number of links connected to the animals or consumers is called the animal distribution. This means that four types of degree distributions – the plant and animal distributions of both the mutualistic and antagonistic (food web) networks – are analyzed here.

The various degree distributions considered here are tested against a maximum entropy (MaxEnt) distribution [29]. The MaxEnt distribution is the probability distribution that maximizes the information entropy subject to a set of information-containing constraints, and so assumes no prior information other than the stated constraints. Here the only information used is the number of nodes in each group of nodes in the bipartite networks and the number of links between the groups.

In the animal distribution, the potential number of links from each animal ranges from 1 to *S_P_* and the mean number of links from each animal is *L*/*S_A_*. In the plant distribution, the potential number of links to each plant ranges from 1 to *S_A_* and the mean number of links to each plant is *L*/*S_P_*. In general, the problem is to find a discrete distribution on a set of *n* values {*x_1_*,…,*x_n_*} (here {1…*S_P_*} or {1…*S_A_*}) with mean *μ* (here *L*/*S_A_* or *L*/*S_P_* respectively) that maximizes 
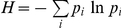
 subject to the constraints 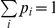 and 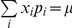. The MaxEnt distribution, found using Lagrange multipliers, is 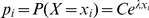 for i = 1,…,*n*; the constants *C* and *λ* are determined by the constraints given above [26], [33].

The problem addressed here is determining the distribution of the number of links attached to each node rather than the exact arrangement of the links, so the system configuration is a vector of *S_A_* or *S_P_* counts, each ranging between 1 and *S_P_* or *S_A_*, rather than a vector of *L* species index pairs, with each index ranging between 1 and *S_A_* or *S_P_* from which a degree distribution could be computed. In the language of a recent study of MaxEnt applied to species distributions [34], this is an unlabeled problem; the MaxEnt solution of the labeled problem gives the random model with a binomial degree distribution. Implicit in this formulation are uninformative prior distributions of the probabilities *p_i_*; the constraint on the mean number of links per node is a soft constraint [34].

Two tests of the fit of the MaxEnt models to the empirical data were used [29]. In the first, the likelihood ratio (*G*) statistic [35] is used to compare an observed distribution to some expected (model) distribution. *G* is defined as 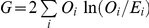 where *O_i_* is the observed frequency, *E_i_* the expected (MaxEnt) frequency and *i* indexes through all values in the discrete distribution with non-zero expected value. A 10,000 trial randomization is used. In each trial, a sample is drawn from the maximum entropy distribution and its *G* value is compared to the *G* value of the empirical distribution. The goodness of fit *f_G_*, is the fraction of trials in which *G* of the empirical distribution is greater than *G* of the sample from the maximum entropy distribution. If *f_G_*<0.95, the empirical network's degree distribution is not significantly different from the model distribution at the 95% confidence level.

The goodness of fit *f_G_* does not differentiate between webs with overly broad or narrow degree distributions. This is measured by the relative width of a distribution *W* = *log*(*σ_O_*/*σ_M_*) where *σ_O_* is the standard deviation of the observed distribution and *σ_M_* is the standard deviation of the model distribution. For each empirical web, the distribution of *W* was computed for 10,000 webs drawn from the model distribution. The quantity *W_95_* is the deviation of the empirical value of *W* from the model median normalized by the width of the upper or lower half of the central interval of the model distribution of *W* at the 95% significance level. Webs with *W_95_*<−1 have distributions that are significantly narrower than the model distributions; *W_95_*>1 occurs for distributions significantly broader than the model distributions.

**Figure 1:**
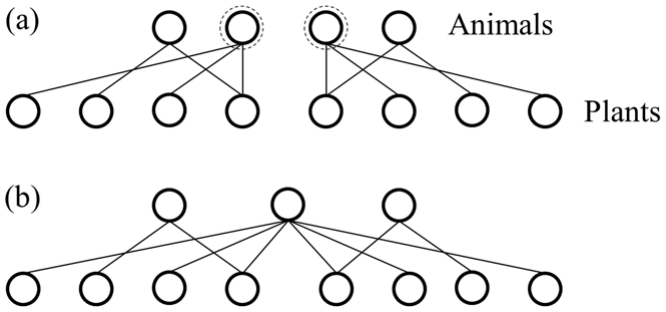
Schematic showing (a) two bipartite networks coupled in (b) by making the most general animal species (marked with a dotted circle in (a)) be the only node shared across the two subwebs.

Some of the larger empirical systems are characterized by strong spatial or temporal heterogeneity, for example a system scattered over several islands with very few species in common across the set of islands. To help understand the degree distributions of these systems, I developed a simple heterogeneous-system degree distribution model in which two identical networks are connected by their most general animal, with every other species unique to each sub-network. I create an animal degree distribution by drawing a sample degree distribution for a sub-system with specified *S_A_*, *S_P_* and *C_B_* that has a MaxEnt degree distribution and then build a new degree distribution by connecting two copies of this sub-system by sharing the most general animal species. This process is illustrated in figure 1 and leads to a final network with 2*S_A_*,- 1 animal species and 2*S_P_* plants

## Results

Using criteria for goodness of fit based on both a likelihood ratio test (*f_G_*<1) and relative width (−1<*W_95_*<1), the MaxEnt model cannot be rejected as a model for the degree distributions of a large fraction of the data sets. Overall, the MaxEnt null model cannot be rejected in 159 (69%) of the 230 degree distributions of 115 networks tested. The MaxEnt model performed equally well on the plant and animal distributions, and cannot be rejected for 81 (70%) of the 115 plant distributions and 78 (68%) of the animal distributions.

None of the antagonistic networks have *S*>134, so the relative performance of the MaxEnt model on mutualistic and antagonistic networks is studied in more detail on the 98 networks with *S*<135; the 17 mutualistic networks with S>140 are studied separately. Table 1 shows that using the criteria of goodness of fit based on both *f_G_* and *W_95_*, the MaxEnt model cannot be rejected in 151 (77%) of the 196 degree distributions from networks with *S*<135. There is clear scale dependence in the fit of the MaxEnt model, with only 8 of 34 (24%) degree distributions of the larger mutualistic networks (*S*>140) well fit by the MaxEnt model.

**Table 1:** Number and (fraction) of networks well-fit by the MaxEnt model for plant and animal degree distributions in networks with *S*<135 and S>140.

	*N*	Plant Distr, Good Fit	Animal Distr, Good Fit
All, S<135	98	73 (0.74)	78 (0.80)
Mutualistic, S<135	51	43 (0.84)	41 (0.80)
Antagonistic, S<135	47	30 (0.64)	37 (0.79)
Mutualistic, S>140	17	8 (0.47)	0

Both *f_G_*<0.95 and −1<*W_95_*<1 are required for the degree distribution to be considered a good fit to the MaxEnt model.

The results in table 1 for the 51 mutualistic and 47 antagonistic networks with *S*<135 show that antagonistic and mutualistic networks display marked differences in their plant degree distributions. While the animal distributions of both network types are equally well predicted by the MaxEnt model, there is asymmetry in the fit of the MaxEnt model to the degree distributions of the number of links to plants. The MaxEnt model cannot be rejected for 84% of the mutualistic network plant distributions, significantly more than the 64% of antagonistic network plant distributions for which the MaxEnt model cannot be rejected (Fisher's Exact test, 2-tailed *p* = 0.02).

**Figure 2:**
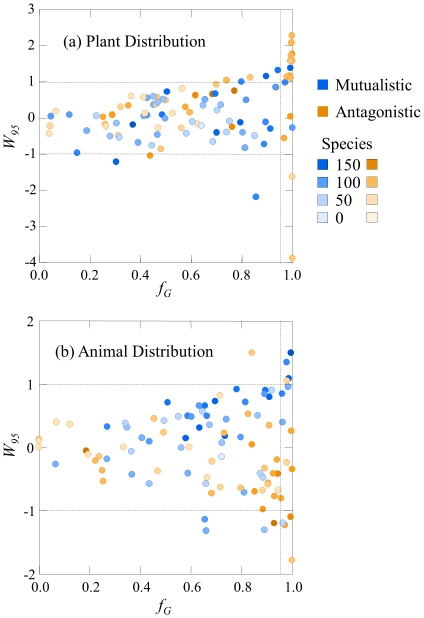
Relative width *W_95_* versus goodness of fit *f_G_* of the MaxEnt model for (a) plant distributions and (b) animal distributions of 98 networks with *S*<135. Shading of the data points shows the number of species in the networks.

Figure 2 further explores the differences in the performance of the MaxEnt model on the different network and degree distribution types for networks with S<135 by plotting *f_G_* versus *W_95_* and coloring the point to show network size. Figure 1a (orange data points) shows that compared to the MaxEnt distributions, the poorly-fit plant distributions of the antagonistic networks tend to be more broadly distributed (13 of 17 have *W_95_*>0, *p* = 0.025, binomial test). There is no significant trend in the width of the poorly-fit plant distributions of the mutualistic networks (fig. 2a, blue) (5 of 8 have *W_95_*>0) or in distribution width (*W_95_*) among the poorly-fit animal distributions of either network type (fig. 2b, 3 of 10 poorly-fit antagonistic networks distributions have *W_95_*>0; 6 of 10 mutualistic network distributions have *W_95_*>0).

**Figure 3:**
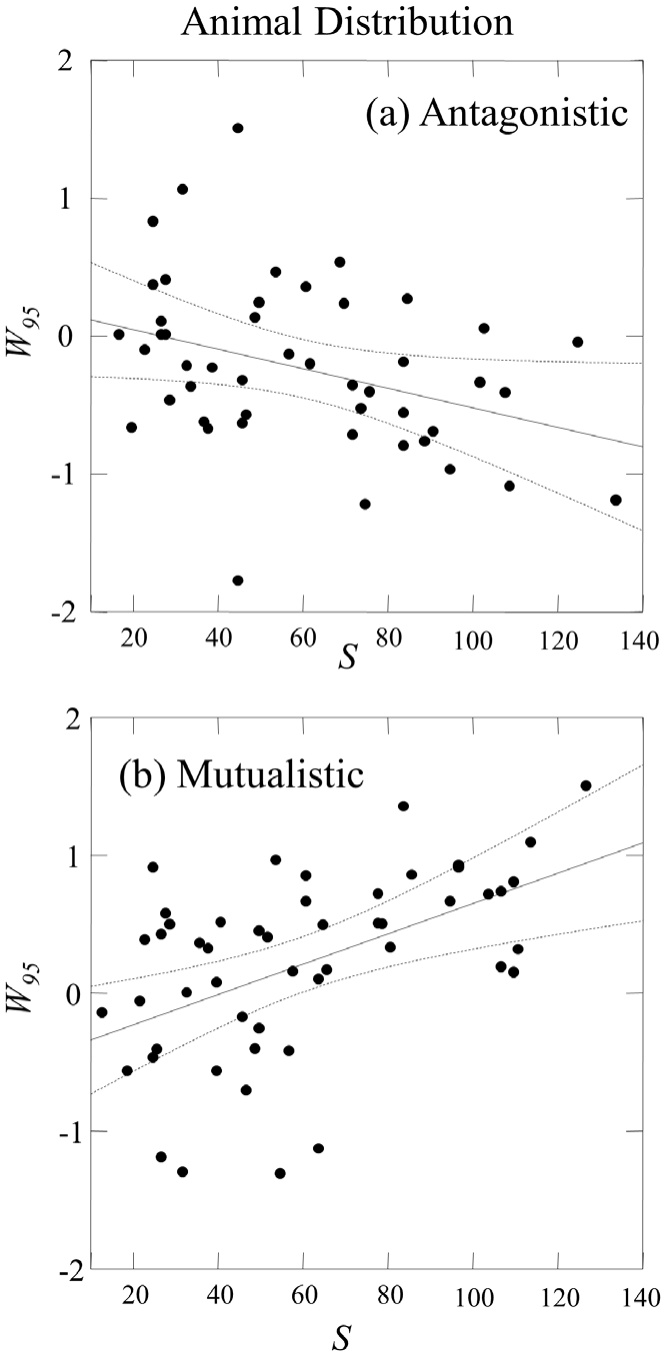
Relative width *W_95_* versus number of species *S* for the animal distributions of (a) antagonistic networks and (b) mutualistic networks with *S*<135. Solid line is linear regression, dotted lines are upper and lower confidence intervals. In (a) *R^2^* = 0.25, *p*<0.001, in (b) *R^2^* = 0.10, *p* = 0.015.

Figure 2b suggests a trend in the width of the animal distributions related to the size of the network. Regressing *W_95_* against *S* shows that there is significant scale dependence in the relative width of the animal distribution in mutualistic and antagonistic networks (fig. 3). As *S* grows, antagonistic network animal distributions are more narrowly distributed than predicted by the MaxEnt model while the animal distributions of the mutualistic networks are more broadly distributed than predicted by the MaxEnt model. No such trends exist in the values of *W_95_* of the plant distributions.

**Figure 4:**
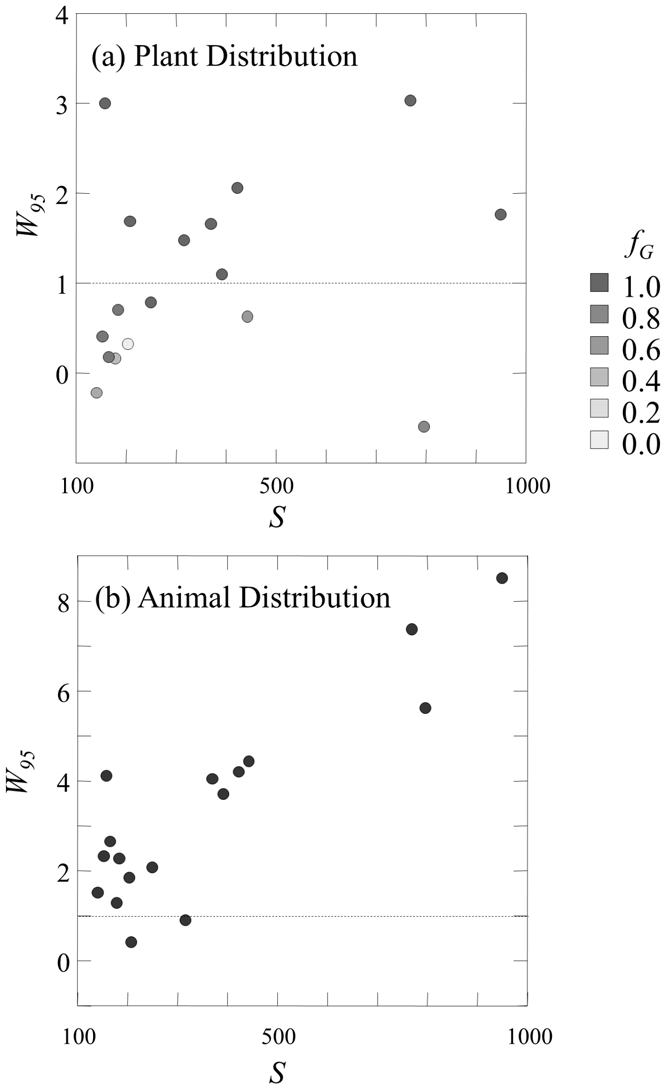
Relative width *W_95_* versus number of species *S* of (a) plant distributions and (b) animal distributions for 17 networks with *S*>140. Shading of data points shows the goodness of fit *f_G_* of the MaxEnt model.

Figure 4 further examines the scale dependence of the fit of the large mutualistic networks to the MaxEnt model. There are 17 mutualistic networks with *S*>140. Most poorly-fit plant and animal distributions are much broader than predicted by the MaxEnt model, with the animal distributions having particularly large values of *W_95_*. The plant distribution of 9 of these is poorly fit by the MaxEnt model based on the dual criteria *f_G_*<0.95 and −1<*W_95_*<1 (fig. 4a). The MaxEnt model is rejected at the 0.95 level for *f_G_* for the animal distribution of all 17 large networks. Only two of these networks have *W_95_*<1 (fig. 4b), and these two networks are the only two seed dispersal networks among the 17 large networks.

I examined two large mutualistic networks in more detail to explore how specific features of these networks might cause their degree distributions to be different from the MaxEnt model. The large network with the most highly anomalous animal distribution, as measured by *W_95_*, is the MULL web. This web is a compilation of previously published data and new observations of plant-insect pollination interactions from across the Galápagos archipelago [36]. Thus these data are from multiple island communities tied together by a common, generalist pollinator. In the MULL web, the dominant pollinator is *Xylocopa darwini*, the Galapagos carpenter bee [37], pollinating 80 of the 105 plants. The next most general pollinator interacts with 14 plants. If this highly general species is removed from the network, the animal distribution becomes much more narrowly distributed, with *W_95_* droping from 14.7 to 1.34. This shows the important role that one species can have in shaping the degree distribution. Remaining deviation from the MaxEnt model is driven by the abundance of highly specialized pollinators – in the MULL web, 31 of 54 species (57%) pollinate a single plant, compared to a range of 15% to 37% (2 S.E. about the mean of 26%) predicted by the MaxEnt model.

The phryganic ecosystem network PTND [38] is another large network with a very broad animal distribution compared to the MaxEnt model, with *W_95_* = 7.41. Part of this is because the system has a dominant pollinator, the European honeybee, *Apis mellifera*, which pollinates 104 of the 131 plant species, while the next most general pollinator interacts with 38 plants. The animal distribution also has a large number of specialists, with 248 of 666 (37%) pollinators specialized on a single plant compared to a range of 20% to 25% (2 S.E. about the mean of 23%) predicted by the MaxEnt model. The system was observed continuously for 50 months. Even with this level of observation effort, pollinator count versus time suggests that the full diversity of the system was not observed. There was also considerable inter-annual variability - during each calendar year, typically about half of the species in any one pollinator group were observed, and only about 20% of pollinators occurred in all years. There is high year-to-year turnover in both the animal and plant communities, and it is likely that some specialists are “apparent specialists”, where the observed specialization is caused by undersampling or sampling in unusual years [39].

These webs have high degrees of spatial (MULL) and temporal (PTND) heterogeneity. I tested the effects of spatial or temporal heterogeneity using the heterogeneous-system degree distribution model, which couples subsystems using a common generalist animal species, on a range of network sizes. The results for *S_A_* = 20, *S_P_* = 20 and *C_B_* = 0.25 are representative of the model's behavior. The MaxEnt model is always rejected as a model for the resulting animal degree distribution of the 79 species (*S_A_* = 39, *S_P_* = 40, *C_B_* = 0.128) network even though the MaxEnt model was used to create the degree distribution of each sub-network. Compared to the MaxEnt model, the networks built by connecting two identical networks are more highly skewed than expected (<*W_95_*> = 2.46, 100 iterations) because of the occurrence of a single highly general pollinator.

## Discussion

While degree distributions in mutualistic and antagonistic networks are strongly skewed, with many species having few connections and few species having many connections, the results here show that their shape can usually be explained by a simple statistical model and does not require a model involving specific ecological or evolutionary processes. The MaxEnt model is found to be a good model of the degree distributions of mutualistic and antagonistic networks more often than it was found to be a good model for food web degree distributions [29], suggesting that ecological processes play a more important role in structuring multi-trophic level food webs than the bipartite networks considered here. Recently, models based on MaxEnt have also been used to explain a broad range of macroecological distributions, such as species-abundance and species area relationships [27], [28], [34]. Together, these findings show that a wide range of large-scale ecological patterns can be explained without turning to detailed descriptions of the ecological processes at work in the system.

An earlier null model for degree distributions in mutualistic networks suggested that species' degree (number of species it interacts with) is a function of its frequency of interaction [11]. Other explanations relate species degree to specific trait combinations making certain links impossible (so-called “forbidden links”) [9], [40] or to a combination of abundance and traits [41]. Evolutionary network models have also been explored as explanations for the structure of ecological networks and a range of degree distributions have been found [42], [43]. These models suggest that the observed exponential-like degree distributions results from variation in the links passed from parent to child species during evolution. A recent analysis of the application of MaxEnt to species abundance distribution argues that it is common for distributions, each resulting from one or more mechanistic model, to also be found as a solution of an appropriately formulated entropy maximization problem [34]. The fact that the formulation used here is so often successful suggests that its formulation and constraints reflect simple constraints commonly operating on these systems. The existence of multiple mechanistic models giving similar degree distributions suggests that multiple mechanisms can place similar simple constraints on the degree distributions, whether through trait distributions or evolutionary processes. This in turn suggests that it will not be possible to determine which ecological or evolutionary processes are constraining the structure of mutualistic networks by studying their degree distributions alone.

When deviations from the MaxEnt model do occur, it is necessary to question whether they are due to ecological processes or systematic sampling biases shaping the degree distributions. I have identified three deviations from the MaxEnt model in the degree distributions of the networks studied here. Importantly, these deviations are different in antagonistic and mutualistic networks, suggesting that different processes at work structuring networks with different types of links. First, plant distributions of the mutualistic networks are significantly better fit by the MaxEnt model than the plant distributions of the antagonistic networks. Second, plant distributions of antagonistic networks tend to be more broadly distributed than predicted by the MaxEnt model. This means that antagonistic networks generally have both more highly vulnerable plant resources and more relatively invulnerable plant resources than predicted by this simple null model.

Third, there are opposite trends in the scale-dependence of the relative width of the animal distributions of mutualistic and antagonistic networks. The animal distribution of large mutualistic networks tends to be more broadly distributed than predicted by the MaxEnt model, while the animal distributions of larger antagonistic networks tend to be more narrowly distributed. Since pollinators and seed dispersers also consume the plants that they benefit reproductively, this suggests that highly generalist animals only occur if they are also conferring a reproductive benefit to their resource. In food webs, it has been suggested that generalist intermediate species are uncommon because of their destabilizing influence on the system [44]. The results presented here suggest that restricted relative generality of plant consumers is more common in larger networks.

There are two sources of deviations from MaxEnt distributions in large mutualistic network animal distributions. First, the degree distribution can be strongly affected by the presence of a single highly connected species, causing a markedly high value of *W_95_*. Second, a larger than predicted fraction of species interacting with a single species can lead to the network having a distribution with a high value of *W_95_*. A detailed examination of two of these data sets helped reveal potential reasons for their broad animal distributions.

A recent simulation study [45] suggests that spatial processes can have important effects on the structure of mutualistic networks, though did not specifically address their degree distributions. The simple heterogeneous-system degree distribution model suggests a biological explanation for the broad degree distributions seen in the large, low connectance pollination networks with a small number of super-generalist pollinators. Strong spatial compartmentalization within sub-networks, leading to networks that contain relatively high connectance sub-networks with MaxEnt degree distributions that are interconnected by one or a small number of highly general pollinators, could lead to the observed highly skewed distributions.

The MULL network also has a large number of animals that pollinate a single plant species. Again, questions arise as to whether this phenomenon is determined by methodology or biology. It could be driven by the relative abundance of the species involved and the observation effort expended [11]. Alternatively, it might be the result of greater than expected specialization of the plant and animals in this system leading to relatively abundant but specialized species.

Given the highly variable phenology of plants and the multiple seasons over which the PTND data [38] were collected, it is likely that the community is functioning as a set of sub-networks separated in time, with specialist pollinators active at different times of year or in different years, connected by common generalist pollinators that are much more regularly present. Here time rather than space is leading to heterogeneity in the community [46], but with a similar effect on the network degree distribution. Other recent studies suggest that strong temporal heterogeneity is a common feature of pollination networks, and so the temporal sampling scheme must be considered when interpreting the relative degree of specialization among species [47], [48].

Analysis of the deviations from the MaxEnt model in these two data sets demonstrates how the MaxEnt model can focus attention on the particular features of degree distributions which require further explanation. Here, it was found that spatial and temporal heterogeneity might play an important role in shaping the degree distributions and other features of the network's structure. This possibility was also highlighted in a number of recent studies [39], [45], [47], [48], [49]. Spatio-temporal heterogeneity is another mechanism which explains why some links cannot occur (“forbidden links”), caused by the lack of species co-occurrence at appropriate points in their life history. Forbidden links are often hypothesized to be an important driver of the structure of mutualistic networks assumed to arise from complementary traits in co-occurring species [9], [40] – here those traits are the spatial or temporal domains in which the species occur. Some large systems are composed of loosely coupled small systems which are either, like MULL, highly spatially heterogeneous or, like PTND, temporally heterogeneous. The observed degree distribution will then depend on an observer's definition of the system's boundaries.

While the MaxEnt null model is useful for understanding how ecosystem features such as spatial and temporal heterogeneity can affect network structure, methodological variability across the available data limit the ecological insight that can be drawn from analyses across a broad range of data sets. As noted in earlier studies, similar limitations driven by variability in data collection protocols still exist in the data describing antagonistic networks [29], [50], [51]. There is a clear need for more consistent data collection protocols and for systematic studies of the effects of variability in data gathering procedures and data collection effort on observed network structure. Despite these issues, the MaxEnt model successfully describes the degree distributions of bipartite ecological networks across a wide range of empirical data. Rather than requiring detailed understanding of the ecological or co-evolutionary processes at work in these systems, the relative abundance of specialist and generalist species in these networks can usually be explained by a simple statistical model.

## Supporting Information

Table S1 **Data Sets Used.** Type: FW = food web; SD = seed dispersal; P = pollination. *S* is the number of taxa, *S_P_* is the number of plant taxa; *S_A_* is the number of animal taxa; *L* is the number of links; *C_B_* = *L/S_B_S_A_* is the bipartite network connectance. Details of sources for food webs are in [29]. Details of sources for mutualistic networks are in [30] and [31] and the data are available at http://ieg.ebd.csic.es/JordiBascompte/Resources.html.
(DOC)
