# Supplementary material for: Biology, Methodology or Chance? The Degree Distributions of Bipartite Ecological Networks
Source: PLoS One. 2011 Mar 3;6(3):e17645. doi: 10.1371/journal.pone.0017645 (PMC3048397; doi:10.1371/journal.pone.0017645)
Supplement: Table S1 — Data Sets Used. Type: FW = food web; SD = seed dispersal; P = pollination. S is the number of taxa, SP is the number of plant taxa; SA is the number of animal taxa; L is the number of links; CB = L/SBSA is the bipartite network connectance. Details of sources for food webs are in [29]. Details of sources for mutualistic networks are in [30] and [31] and the data are available at http://ieg.ebd.csic.es/JordiBascompte/Resources.html. (DOC) [file pone.0017645.s001.doc]

Supporting Information for “Biology, Methodology or Chance? The Degree Distributions of Bipartite Ecological Networks” by Richard J. Williams

Table S1. Data Sets Used. Type: FW=food web; SD=seed dispersal; P=pollination. *S* is the number of taxa, *S_P_* is the number of plant taxa; *S_A_* is the number of animal taxa; *L* is the number of links; *C_B_* = *L/S_B_S_A_* is the bipartite network connectance. Details of sources for food webs are in (Williams 2010). Details of sources for mutualistic networks are in (Bascompte et al. 2006) and (Rezende et al. 2007) and the data are available at http://ieg.ebd.csic.es/JordiBascompte/Resources.html .

| Web | Type | *S* | *S_P_* | *S_A_* | *L* | *C_B_* |
| --- | --- | --- | --- | --- | --- | --- |
| AkatoreA | FW | 84 | 43 | 41 | 210 | 0.11912 |
| AkatoreB | FW | 54 | 26 | 28 | 95 | 0.13049 |
| Berwick | FW | 72 | 35 | 37 | 216 | 0.1668 |
| Blackrock | FW | 84 | 49 | 35 | 338 | 0.19708 |
| Broad | FW | 91 | 53 | 38 | 524 | 0.26018 |
| Canton | FW | 108 | 56 | 52 | 660 | 0.22665 |
| Coachella | FW | 25 | 3 | 22 | 33 | 0.5 |
| CompanyBay | FW | 47 | 3 | 44 | 62 | 0.4697 |
| Coweeta17 | FW | 70 | 38 | 32 | 128 | 0.10526 |
| Coweeta1 | FW | 57 | 28 | 29 | 103 | 0.12685 |
| Dempsters | FW | 102 | 50 | 52 | 792 | 0.30462 |
| DuffinCreek | FW | 45 | 32 | 13 | 168 | 0.40384 |
| ElVerde | FW | 125 | 28 | 97 | 248 | 0.091311 |
| German | FW | 84 | 48 | 36 | 298 | 0.17245 |
| KyeBurn | FW | 95 | 58 | 37 | 582 | 0.2712 |
| Lerderderg | FW | 39 | 9 | 30 | 86 | 0.31852 |
| LittleKyeBurn | FW | 75 | 42 | 33 | 315 | 0.22727 |
| Martins | FW | 103 | 48 | 55 | 262 | 0.099243 |
| Mimihau | FW | 62 | 30 | 32 | 158 | 0.16458 |
| Narrowdale | FW | 69 | 28 | 41 | 127 | 0.11063 |
| NorthCol | FW | 76 | 25 | 51 | 188 | 0.14745 |
| Powder | FW | 74 | 32 | 42 | 221 | 0.16444 |
| Stony | FW | 109 | 63 | 46 | 772 | 0.26639 |
| Sutton | FW | 85 | 63 | 22 | 407 | 0.29365 |
| Troy | FW | 72 | 40 | 32 | 144 | 0.1125 |
| Wisp | FW | 46 | 14 | 32 | 89 | 0.19866 |
| Gold | FW | 34 | 6 | 28 | 46 | 0.27381 |
| Havens | FW | 23 | 8 | 15 | 34 | 0.28333 |
| Hawkins | FW | 28 | 10 | 18 | 19 | 0.10556 |
| Healy | FW | 89 | 47 | 42 | 539 | 0.27305 |
| LittleRock | FW | 61 | 12 | 49 | 93 | 0.15816 |
| Reef | FW | 38 | 3 | 35 | 57 | 0.54286 |
| Shelf | FW | 46 | 3 | 43 | 54 | 0.4186 |
| Stmarks | FW | 37 | 6 | 31 | 55 | 0.2957 |
| Ulan | FW | 20 | 5 | 15 | 23 | 0.30667 |
| web106 | FW | 29 | 5 | 24 | 35 | 0.29167 |
| web123 | FW | 134 | 98 | 36 | 355 | 0.10062 |
| web207 | FW | 32 | 17 | 15 | 120 | 0.47059 |
| web210 | FW | 45 | 7 | 38 | 94 | 0.35338 |
| web28 | FW | 27 | 7 | 20 | 25 | 0.17857 |
| web33 | FW | 28 | 5 | 23 | 27 | 0.23478 |
| web38 | FW | 25 | 3 | 22 | 30 | 0.45455 |
| web39 | FW | 27 | 5 | 22 | 32 | 0.29091 |
| web59 | FW | 17 | 6 | 11 | 15 | 0.22727 |
| web99 | FW | 33 | 9 | 24 | 44 | 0.2037 |
| Ythan | FW | 50 | 7 | 43 | 54 | 0.1794 |
| Ythannew | FW | 49 | 5 | 44 | 51 | 0.23182 |
| Arr1 | P | 185 | 84 | 101 | 361 | 0.042551 |
| Arr2 | P | 107 | 43 | 64 | 196 | 0.071219 |
| Arr3 | P | 61 | 36 | 25 | 81 | 0.089999 |
| Bahe | P | 114 | 12 | 102 | 167 | 0.13644 |
| Cllo | P | 371 | 96 | 275 | 923 | 0.034962 |
| Dihi | P | 78 | 17 | 61 | 146 | 0.14079 |
| Dish | P | 52 | 16 | 36 | 85 | 0.14757 |
| Dupo | P | 49 | 11 | 38 | 106 | 0.25359 |
| Eol | P | 142 | 24 | 118 | 242 | 0.085455 |
| Eolz | P | 107 | 31 | 76 | 456 | 0.19355 |
| Eski | P | 27 | 14 | 13 | 52 | 0.28572 |
| Herr | P | 205 | 26 | 179 | 412 | 0.088526 |
| Hock | P | 110 | 29 | 81 | 179 | 0.076201 |
| Inpk | P | 127 | 42 | 85 | 269 | 0.07535 |
| Kevn | P | 111 | 20 | 91 | 190 | 0.1044 |
| Kt90 | P | 770 | 91 | 679 | 1193 | 0.019307 |
| Med1 | P | 66 | 21 | 45 | 83 | 0.08783 |
| Med2 | P | 95 | 23 | 72 | 125 | 0.075481 |
| Memm | P | 104 | 25 | 79 | 299 | 0.15139 |
| Moma | P | 29 | 11 | 18 | 38 | 0.19192 |
| Mott | P | 57 | 13 | 44 | 143 | 0.25 |
| Mull | P | 159 | 105 | 54 | 204 | 0.035979 |
| Oflo | P | 22 | 10 | 12 | 30 | 0.25 |
| Ofst | P | 50 | 8 | 42 | 79 | 0.23512 |
| Olau | P | 84 | 29 | 55 | 145 | 0.09091 |
| Olle | P | 65 | 9 | 56 | 103 | 0.20437 |
| P11_Hestehaven-B | P | 50 | 10 | 40 | 72 | 0.18 |
| P13_Hestehaven-W | P | 110 | 28 | 82 | 250 | 0.10888 |
| P19_Yamazaki&Kato | P | 393 | 99 | 294 | 588 | 0.020202 |
| P4_Inoue_etal | P | 951 | 112 | 839 | 1875 | 0.019954 |
| P6_Kakutani_etal | P | 424 | 112 | 314 | 768 | 0.021838 |
| P7_Kato&Miura1996 | P | 251 | 64 | 187 | 430 | 0.035929 |
| P9_Kato_etal1993 | P | 444 | 90 | 354 | 865 | 0.02715 |
| Perc | P | 97 | 61 | 36 | 178 | 0.081056 |
| Prap | P | 78 | 18 | 60 | 120 | 0.11111 |
| Prca | P | 180 | 41 | 139 | 374 | 0.065624 |
| Prcg | P | 167 | 49 | 118 | 346 | 0.059839 |
| Ptnd | P | 797 | 131 | 666 | 2933 | 0.033618 |
| Rabr | P | 81 | 28 | 53 | 109 | 0.073449 |
| Rmrz | P | 97 | 48 | 49 | 156 | 0.066327 |
| Schm | P | 40 | 7 | 33 | 65 | 0.28139 |
| Smal | P | 47 | 13 | 34 | 141 | 0.31901 |
| Smra | P | 154 | 26 | 128 | 312 | 0.093752 |
| Bair | SD | 28 | 7 | 21 | 50 | 0.34014 |
| Beeh | SD | 40 | 31 | 9 | 119 | 0.42652 |
| Cacg | SD | 41 | 25 | 16 | 68 | 0.17 |
| Caci | SD | 54 | 34 | 20 | 95 | 0.13971 |
| Caco | SD | 38 | 25 | 13 | 49 | 0.15077 |
| Cafr | SD | 36 | 21 | 15 | 51 | 0.16191 |
| Crom | SD | 79 | 72 | 7 | 143 | 0.28373 |
| Fros | SD | 26 | 16 | 10 | 110 | 0.68749 |
| Gen1 | SD | 25 | 7 | 18 | 38 | 0.30159 |
| Gen2 | SD | 64 | 35 | 29 | 146 | 0.14384 |
| Hamm | SD | 55 | 36 | 19 | 197 | 0.28801 |
| Hrat | SD | 33 | 16 | 17 | 121 | 0.44485 |
| Kant | SD | 32 | 5 | 27 | 86 | 0.63703 |
| Lamb | SD | 86 | 25 | 61 | 511 | 0.33508 |
| Lope | SD | 27 | 19 | 8 | 75 | 0.49342 |
| Mack | SD | 61 | 29 | 32 | 66 | 0.07112 |
| Mont | SD | 209 | 169 | 40 | 666 | 0.098521 |
| Ncor | SD | 58 | 25 | 33 | 154 | 0.18667 |
| Nnog | SD | 46 | 18 | 28 | 129 | 0.25595 |
| SD2_Sorensen | SD | 13 | 7 | 6 | 22 | 0.52382 |
| SD4_Guitian | SD | 19 | 12 | 7 | 40 | 0.47618 |
| Sapf | SD | 23 | 15 | 8 | 38.001 | 0.31667 |
| Snow | SD | 64 | 50 | 14 | 234 | 0.33429 |
| Wes | SD | 317 | 207 | 110 | 1121 | 0.049229 |
| Wyth | SD | 25 | 11 | 14 | 47 | 0.30519 |

Bascompte J, Jordano P, Olesen JM (2006) Asymmetric Coevolutionary Networks Facilitate Biodiversity Maintenance. Science 312: 431 - 433.

Rezende EL, Lavabre JE, Guimarães PR, Jordano P, Bascompte J (2007) Non-random coextinctions in phylogenetically structured mutualistic networks. Nature 448: 925-928.

Williams RJ (2010) Simple MaxEnt models explain food web degree distributions. Theoretical Ecology 3: 45-52.
